# Photocatalytic Removal of Microcystin-LR by Advanced WO_**3**_-Based Nanoparticles under Simulated Solar Light

**DOI:** 10.1155/2015/720706

**Published:** 2015-03-25

**Authors:** Chao Zhao, Dawei Li, Yonggang Liu, Chuanping Feng, Zhenya Zhang, Norio Sugiura, Yingnan Yang

**Affiliations:** ^1^Graduate School of Life and Environmental Sciences, University of Tsukuba, Tsukuba 305-8572, Japan; ^2^School of Water Resources and Environment, China University of Geosciences, Beijing 100083, China; ^3^Institute of Environmental Sciences, Zhengzhou University, Zhengzhou 450001, China

## Abstract

A series of advanced WO_3_-based photocatalysts including CuO/WO_3_, Pd/WO_3_, and Pt/WO_3_ were synthesized for the photocatalytic removal of microcystin-LR (MC-LR) under simulated solar light. In the present study, Pt/WO_3_ exhibited the best performance for the photocatalytic degradation of MC-LR. The MC-LR degradation can be described by pseudo-first-order kinetic model. Chloride ion (Cl^−^) with proper concentration could enhance the MC-LR degradation. The presence of metal cations (Cu^2+^ and Fe^3+^) improved the photocatalytic degradation of MC-LR. This study suggests that Pt/WO_3_ photocatalytic oxidation under solar light is a promising option for the purification of water containing MC-LR.

## 1. Introduction

Eutrophication in superficial freshwater bodies induced frequent cyanobacteria blooms worldwide. The occurrence of toxic cyanobacterial blooms in eutrophic lakes, reservoirs, and other recreational water has been identified as an increasingly serious problem in many countries [1]. The toxins released into freshwater by cyanobacteria are well documented [2].

Microcystins (MCs) are the most commonly occurring toxins released by cyanobacteria. MCs are cyclic heptapeptides containing the unique C_20_ amino acid, 3-amino-9-methoxy-2,6,8-trimethyl-10-phenyldeca-4,6-dienoic acid (ADDA). MCs are strongly hepatotoxic because they inhibit serine/threonine protein phosphatases 1 and 2A [3]. Acute exposure may result in hepatic injury, promote primary liver cancer, and even cause the death of animals and humans. One of the most common occurring MCs is the highly toxic microcystin-LR (MC-LR), which has leucine (L) and arginine (R) in the variable positions. The World Health Organization (WHO) has determined a provisional guideline value of 1.0 *μ*g L^−1^ for MC-LR in drinking water. Various water treatment processes have been evaluated to determine their performance in decomposing these toxins. However, MCs are chemically stable across a range of pH values and temperatures, due to their cyclic structure. Consequently, traditional water treatment processes are not reliable for the removal of MCs [4–6].

Photocatalytic oxidation as an advanced oxidation technology has been considered an environment-friendly water treatment method [7–11]. When the photocatalyst exposure to a light with appropriate wavelength happens, electron (e^−^) and hole (h^+^) pairs are generated on the catalyst surface. The photogenerated electrons and holes react with oxygen and water molecules or hydroxyl groups adsorbed on photocatalyst surface to form highly reactive species, such as superoxide radicals (∙O_2_^−^) and hydroxyl radicals (∙OH) [12]. These radicals can oxidize a number of organic pollutants including dyes, pesticides, and herbicides [7–11, 13]. Previous research proved that photocatalytic oxidation with TiO_2_ photocatalyst could effectively destroy MCs, even at extremely high toxin concentrations [14, 15]. However, TiO_2_ has a large band gap energy (*E*_*g*_) of 3.2 eV that restricts the wide use of this photocatalyst, because it can only absorb UV light which accounts for 5% of the solar light [16]. Many efforts have been made to enhance the photocatalytic performance of TiO_2_ under solar light. For example, Ag-modified TiO_2_ thin film was developed for bacteria disinfection under solar light [17, 18]. TiO_2_-film/CuO microgrid heterojunction and P-doped TiO_2_ nanoparticles were synthesized for the decomposition of organic dye [19, 20]. By contrast, tungsten trioxide (WO_3_) can utilize solar light more effectively than TiO_2_, because it has an *E*_*g*_ between 2.4 and 2.8 eV [10]. In addition, WO_3_ is inexpensive to prepare and stable in acidic and oxidative conditions, which makes it a promising material for photocatalytic applications. Previous research showed that photocatalytic degradation of organic pollutants such as organic dyes using WO_3_ under solar light was intensified by the presence of suitable dopants, such as Pt, Pd, and CuO [21–23]. However, there is little research on the photocatalytic degradation of MCs using WO_3_-based photocatalysts under solar light.

In the present study, three types of WO_3_-based photocatalysts including CuO/WO_3_, Pd/WO_3_, and Pt/WO_3_ were synthesized for photocatalytic degradation of MC-LR under simulated solar light. The characteristics of developed WO_3_-based photocatalysts were analyzed by BET surface area, X-ray diffraction (XRD), and scanning electron microscopy (SEM). A series of batch experiments were carried out to evaluate the photocatalytic performance of the developed photocatalysts for MC-LR degradation under simulated solar light. On the other hand, chlorides and metal cations are common in water, and they are important in many treatment technologies such as breakpoint chlorination and electrochemical oxidation methods [24]. Therefore, in this study, the effects of chloride ions (Cl^−^) and metal cations (Cu^2+^ and Fe^3+^) on the photocatalytic degradation of MC-LR under solar light were also investigated.

## 2. Experimental

### 2.1. Reagents

WO_3_ powder, microcystin-LR (MC-LR) standard (≥95% purity; FW 995.2 g mol^−1^), terephthalic acid 99%, and Cu(NO_3_)_2_·3H_2_O (99.9% purity) were purchased from Wako (Wako Pure Chemical Industries, Ltd., Japan). Hexachloroplatinic acid (H_2_PtCl_6_·6H_2_O) and Pd powder with a surface area of 40–60 m^2^ g^−1^ were supplied by Sigma-Aldrich (Sigma-Aldrich Co. LLC., USA).

### 2.2. Photocatalyst Preparation

The WO_3_ loaded with 0.1 wt% CuO (marked as CuO/WO_3_) was synthetized by an impregnation method [21]: Cu(NO_3_)_2_ aqueous solution was mixed with WO_3_ powder, and the mixture was dried on hot plate and then calcined at 300°C for 30 min in air.

The Pd doped WO_3_ photocatalyst (Pd/WO_3_) was prepared by the mechanical mixing of Pd (wt% versus WO_3_) and WO_3_ in a ceramic mortar [25].

The Pt modified WO_3_ sample (Pt/WO_3_) was developed using a photodeposition method [21] from H_2_PtCl_6_·6H_2_O on the fine particulate WO_3_ under visible light irradiation in pure water and subsequently in an aqueous methanol (10 vol.%) solution.

### 2.3. Photocatalyst Characterization

The crystalline phases of the prepared photocatalysts were determined using a powder X-ray diffraction (XRD) (Rigaku RINT2200, Japan). The morphology of the prepared photocatalysts was analyzed by a scanning electron microscopy (SEM) (JEOL, JSM-5600, Japan). The specific surface area of the prepared photocatalyst was measured using a BET surface area analyzer (Coulter SA3100, USA).

### 2.4. Photocatalytic Removal of MC-LR

The photocatalytic degradation of MC-LR by prepared WO_3_-based photocatalyst was performed in a 6 mL glass vessel placed on a magnetic stirrer. A simulated solar lamp (XC-100B, SERIC Ltd., Japan) equipped axially at the center region above the glass vessel was employed as the irradiation source. In the present experiments, pure WO_3_ and the developed three types of WO_3_-based photocatalysts (CuO/WO_3_, Pd/WO_3_, and Pt/WO_3_) were previously dispersed in water using an ultrasonic bath sonicator for 30 min. Then the photocatalysts dispersed solutions were transferred to the glass vessels containing MC-LR to obtain a final volume of 5 mL. The initial MC-LR concentration in each glass vessel was 1 mg L^−1^. Before irradiation, the suspension was magnetically stirred for 60 min in the dark to achieve adsorption equilibrium. After that, the lamp was switched on to initiate the photocatalytic reaction. Temperature of the whole laboratory was controlled at 25 ± 1°C by an air conditioner. In addition, a mini-air-circulator was also employed near to the reactor to make sure constant local temperature during the photocatalytic reaction exists. During irradiation, 0.25 mL of sample was withdrawn at a time interval of 30 min, centrifuged at 10000 rpm for 10 min, and filtered through a 0.22 *μ*m filter membrane before the HPLC analysis.

The concentration of MC-LR was measured using a high performance liquid chromatography (HPLC) (Jasco-1500, Jasco, Japan) equipped with a high-resolution diode array detector (Jasco UV-1570) set at 238 nm. Samples were separated on a C18 column (4.6 × 250 mm, 5 *μ*m) using a mixture of acetonitrile and 0.05 M phosphate buffer (pH 6.8; 32 : 68 v/v) as the mobile phase at a flow rate of 1 mL min^−1^. All the experiments were replicated three times under the same conditions and the average value was used for analyses.

### 2.5. Detection of Hydroxyl Radicals (∙OH)

The detection of hydroxyl radicals generated by the prepared WO_3_-based photocatalysts was carried out according to Ishibashi et al. [26]. Terephthalic acid was used as a probe molecule to detect the photogenerated ∙OH radicals in the photocatalytic reaction system. A sample of 4 mg developed WO_3_-based photocatalyst powder was dispersed in a 20 mL solution made of terephthalic acid at 5 × 10^−4^ M dissolved in a 2 × 10^−3^ M NaOH aqueous solution. The simulated solar light was used as an irradiation source. During the irradiation, samples were withdrawn and centrifuged at a 20 min time interval. Then, the centrifuged solution was transferred in a quartz cell and the photoluminescence spectra of 2-hydroxyterephthalic acid generated by the reaction of terephthalic acid with ∙OH were measured on a Hitachi F-4500 fluorescence spectrophotometer. The spectra were recorded between 350 and 550 nm under an excitation at 315 nm.

## 3. Results and Discussion

### 3.1. Characterization of WO_3_-Based Photocatalysts

**Figure 1:**
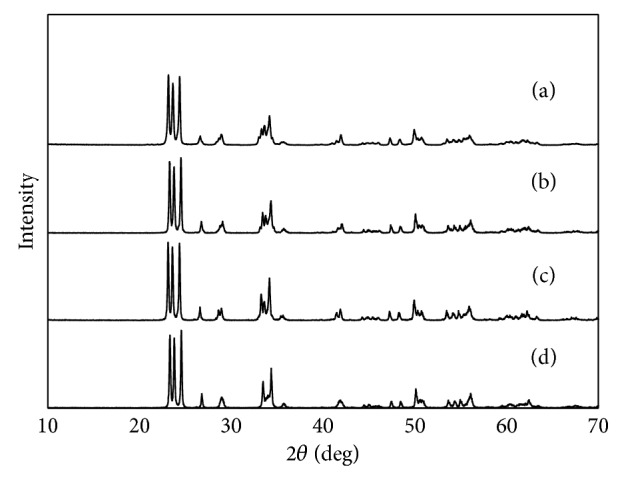
XRD patterns of WO_3_ and modified WO_3_ samples: (a) Pd/WO_3_, (b) Pt/WO_3_, (c) CuO/WO_3_, and (d) pure WO_3_.

The crystalline phases of the developed WO_3_-based photocatalysts were measured by a powder X-ray diffraction (XRD) (Rigaku RINT2200, Japan). Characteristic peaks are observed for all diffraction patterns, which are indexed to the standard card (JCPDS 43-1035). As shown in Figure 1, all samples have monoclinic WO_3_ structure and the metal doping does not influence the crystal structures of WO_3_. No extra peaks except for monoclinic WO_3_ are observed (Figure 1). This phenomenon can be explained by the small amount of CuO, Pd, and Pt species content and high dispersion in the samples.

**Figure 2:**
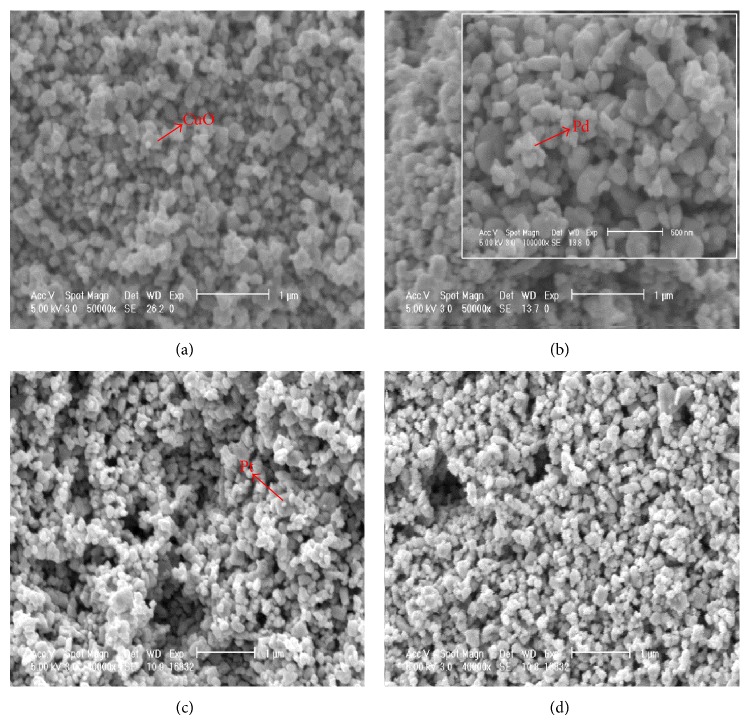
SEM images of WO_3_ and modified WO_3_ samples: (a) CuO/WO_3_, (b) Pd/WO_3_, (c) Pt/WO_3_, and (d) pure WO_3_.

The morphology and microstructure of the developed photocatalysts were analyzed by scanning electron microscopy (SEM) (Figure 2). The SEM images of pure and modified WO_3_ photocatalysts showed that they are composed of particles with size ranging from 100 to 200 nm. The specific surface area of the pure WO_3_ was about 5 m^2^ g^−1^ which is in agreement with other reports [22]. The specific surface area of modified WO_3_ photocatalysts (CuO/WO_3_, Pd/WO_3_, and Pt/WO_3_) was slightly increased to 6.0, 6.5, and 7.0 m^2^ g^−1^, respectively, due to the metals loading and a grind of WO_3_ powders in the preparation process.

### 3.2. Photocatalytic Removal of MC-LR Using Various WO_3_-Based Photocatalysts

**Figure 3:**
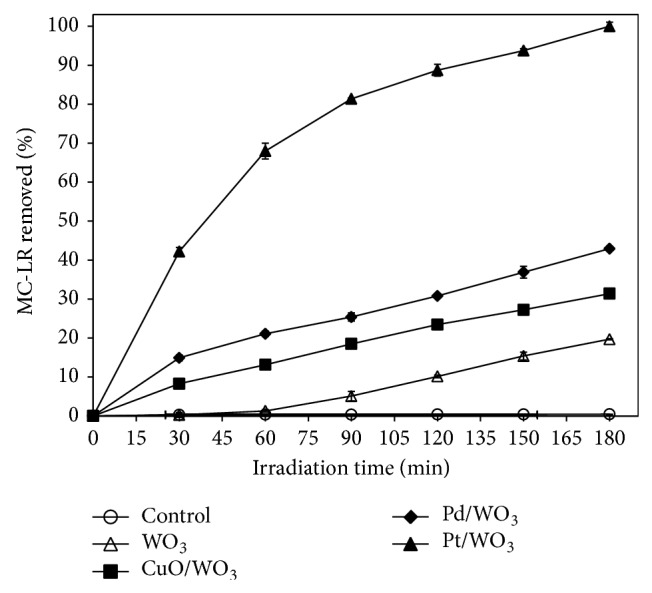
The effect of catalysts on the efficiency of photocatalytic degradation of MC-LR. (Experimental conditions: MC-LR concentration of 1 mg L^−1^, catalyst concentration of 100 mg L^−1^, and simulated solar light intensity of 0.4 mW cm^−2^.)

As shown in Figure 3, the concentration of MC-LR was virtually unchanged after 180 min solar light irradiation when there was no photocatalyst in the solution. This result indicated that MC-LR was stable under solar light irradiation. After 180 min photocatalysis, approximately 24.8% of MC-LR was removed from the aqueous solution in which only pure WO_3_ was added. The modified WO_3_-based photocatalysts (CuO/WO_3_ and Pd/WO_3_) achieved 31.4% and 42.9% MC-LR removal, respectively. The Pt/WO_3_ composite achieved a 100% degradation of MC-LR after 180 min solar light irradiation. The modified WO_3_-based photocatalysts are supported by many previous researches. Arai et al. reported that the photocatalytic activity of Pd/WO_3_ was 2 times higher than that of CuO/WO_3_ in the degradation of acetaldehyde, and the performance of Pt/WO_3_ was better than that of CuO/WO_3_ for decomposing formaldehyde [22, 23]. In this present study, Pt/WO_3_ exhibits the best photocatalytic performance for the degradation of MC-LR under solar light irradiation.

### 3.3. The Mechanism of MC-LR Degradation by WO_3_-Based Photocatalysts

The relative more positive conduction band level of WO_3_ (+0.5 V versus NHE) compared to potential for the single-electron reduction of oxygen (O_2_/O_2_^−^ = −0.56 V versus NHE; O_2_/HO_2_ = −0.13 V versus NHE) was the main reason for the relative slow reaction rate of WO_3_-induced photocatalytic reactions. In the presence of CuO, Pd, and Pt, the reduction of O_2_ molecules can be promoted effectively by a multielectron process (O_2_/H_2_O_2_ = +0.68 V versus NHE; O_2_/H_2_O = +1.23 V versus NHE) [22, 27]. In a photocatalytic reaction, the following chain reactions have been postulated:

(1) $$\begin{array}{c} \text{Catalyst}+hv⟶{\text{e}}^{-}+{\text{h}}^{+} \end{array}$$

(2) $$\begin{array}{c} {\text{O}}_{2}+2{\text{e}}^{-}+2{\text{H}}^{+}⟶{\text{H}}_{2}{\text{O}}_{2} \end{array}$$

(3) $$\begin{array}{c} {\text{H}}_{2}{\text{O}}_{2}⟶2∙\text{OH} \end{array}$$

(4) $$\begin{array}{c} {\text{H}}_{2}\text{O}+{\text{h}}^{+}⟶∙\text{OH}+{\text{H}}^{+} \end{array}$$

**Figure 4:**
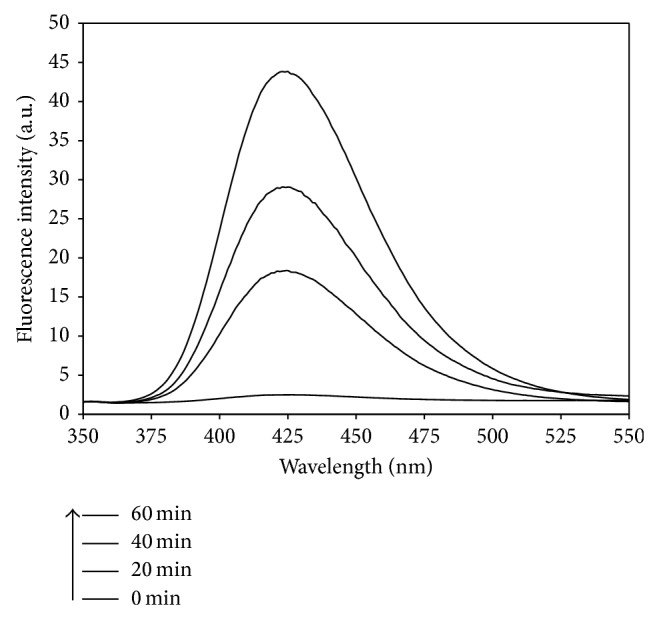
Photoluminescence spectral changes observed during irradiation of the Pt/WO_3_ sample. (Experimental conditions: NaOH concentration of 2 × 10^−3^ M, terephthalic acid concentration of 5 × 10^−4^ M, Pt/WO_3_ concentration of 200 mg L^−1^, and simulated solar light intensity of 0.4 mW cm^−2^.)

Photocatalytic degradation of MC-LR was initiated by the attack of hydroxyl radical (∙OH) on the conjugated diene structure of ADDA [28], indicating the primary reactive species in MC-LR degradation is ∙OH radical. The photogenerated ∙OH radicals can be detected by photoluminescence spectra analysis.  Figure 4 shows the photoluminescent spectral changes of Pt/WO_3_ during 60 min solar light irradiation. At the wavelength of 425 nm, the photoluminescence intensity gradually increased from 2.5 to 43.8 a.u with increasing the irradiation time to 60 min, indicating that ∙OH radicals were generated on the photocatalyst-water interface via photocatalytic reactions [26, 27].

**Figure 5:**
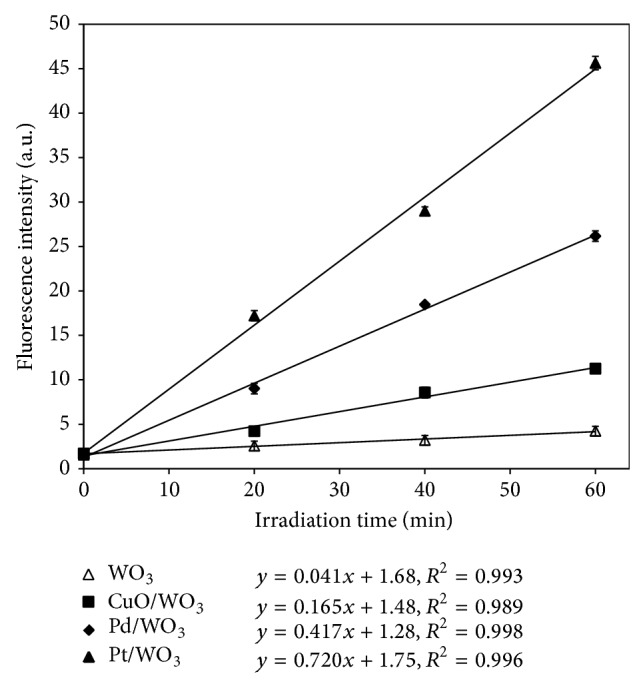
Photoluminescence intensity of pure WO_3_ and modified WO_3_-based photocatalysts as a function of irradiation time. (Experimental conditions: NaOH concentration of 2 × 10^−3^ M, terephthalic acid concentration of 5 × 10^−4^ M, catalyst concentration of 200 mg L^−1^, and simulated solar light intensity of 0.4 mW cm^−2^.)

Figure 5 presents the photoluminescence intensity of pure WO_3_ and modified WO_3_-based photocatalysts at 425 nm as a function of irradiation time. The photoluminescence intensity induced by simulated solar light in terephthalic acid solution was linearly related to the irradiation time. The number of ∙OH radicals generated on the surface of these photocatalysts was proportional to the irradiation time and followed zero-order kinetic model [26, 28]. Furthermore, the slopes of the regression lines represent the generation rate of ∙OH radicals (Figure 5). Without a dopant, WO_3_ could only generate a small number of ∙OH radicals under solar light irradiation. The generation rate of ∙OH radicals on the surface of pure WO_3_ is merely 0.04 a.u min^−1^. When doped with CuO, Pd, and Pt, the generation rate of ∙OH radicals on WO_3_ surface was obviously enhanced. During 60 min solar light irradiation, Pt/WO_3_ achieved the highest generation rate (0.72 a.u min^−1^) of ∙OH radicals, which was much higher than those by CuO/WO_3_ (0.17 a.u min^−1^) and Pd/WO_3_ (0.42 a.u min^−1^). Since the photocatalytic degradation of MC-LR was initiated by the attack of ∙OH radical, Pt/WO_3_ seems to be the most promising photocatalyst for MC-LR removal due to its higher generation rate of ∙OH radicals. Therefore, in the following part, Pt/WO_3_ was selected as the photocatalyst for MC-LR removal under simulated solar light irradiation.

### 3.4. Kinetic Analysis in a Range of Light Intensities

The kinetics of photocatalytic oxidation for MC-LR were analyzed using Langmuir-Hinshelwood (L-H) model expressed as follows:

(5) $$\begin{array}{c} r=\frac{dC}{dt}=\frac{kKC}{\left(1+KC\right)}. \end{array}$$

Since *KC* is much less than 1, if neglecting the term of *KC*, the L-H model can be simplified to a pseudo-first-order kinetic equation:

(6) $$\begin{array}{c} \ln\left(\frac{C_{0}}{C}\right)=kKt=k_{\text{app}}t, \end{array}$$

where *r* is the reaction rate (mg L^−1^ min^−1^), *C*_0_ is the initial concentration of MC-LR after dark adsorption (mg L^−1^), *C* is the concentration of MC-LR at time *t* (mg L^−1^), *t* is the irradiation time (min), *k* is the reaction rate constant (min^−1^), *K* is the adsorption coefficient of MC-LR on a photocatalyst particle (L mg^−1^), and *k*_app_ is the apparent rate constant for the photocatalytic degradation of MC-LR.

**Figure 6:**
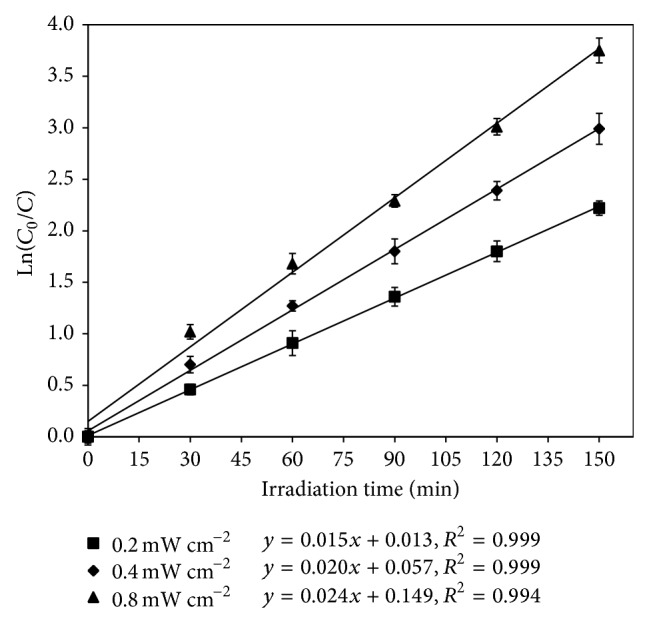
Efficiency of photocatalytic degradation of MC-LR as a function of light intensity. (Experimental conditions: MC-LR concentration of 1 mg L^−1^ and Pt/WO_3_ concentration of 100 mg L^−1^.)

The kinetic curves for the degradation of MC-LR by Pt/WO_3_ under various intensities of solar light irradiation are shown in Figure 6. The correlation coefficient (*R* ^2^) values of linear regression in all the cases are greater than 0.99, which confirms the photocatalytic degradation of MC-LR by Pt/WO_3_ under simulated solar light well follows the pseudo-first-order kinetic equation. The corresponding *k*_app_ values of MC-LR degradation were 0.148, 0.196, and 0.241 min^−1^ under 0.2, 0.4, and 0.8 mW cm^−2^ solar light irradiation, respectively. At higher intensity of solar irradiation, more electron-hole pairs were expected to generate on photocatalyst surface, resulting in the enhancement of MC-LR degradation. According to Ohko et al. [29], if photocatalytic reaction proceeded under purely light-limited conditions, the degradation rate would depend on adsorbed photon numbers (light intensity) linearly. In this present study, a nonlinear relationship of photodegradation rate with light intensity was observed (figure was not shown) that seemingly implies the photocatalytic reaction proceeded under a light-rich condition. In that case, the surface adsorptive property of photocatalyst has a major influence on the photodegradation rate. Although MC-LR concentration showed a very slight decrease during 60 min dark adsorption (removal rate was less than 5%), a systematic study on the effects of initial MC-LR concentration should be carried out in the future research. That is helpful to understand clearly that the photodegradation proceeds under light-rich or light-limited condition. Since the average intensity of natural solar light is generally 0.8 mW cm^−2^, Pt/WO_3_ appears to be a promising photocatalyst for the degradation of MC-LR in practical water.

### 3.5. Effect of Chloride Ion (Cl^−^) on the Photocatalytic Degradation of MC-LR

**Figure 7:**
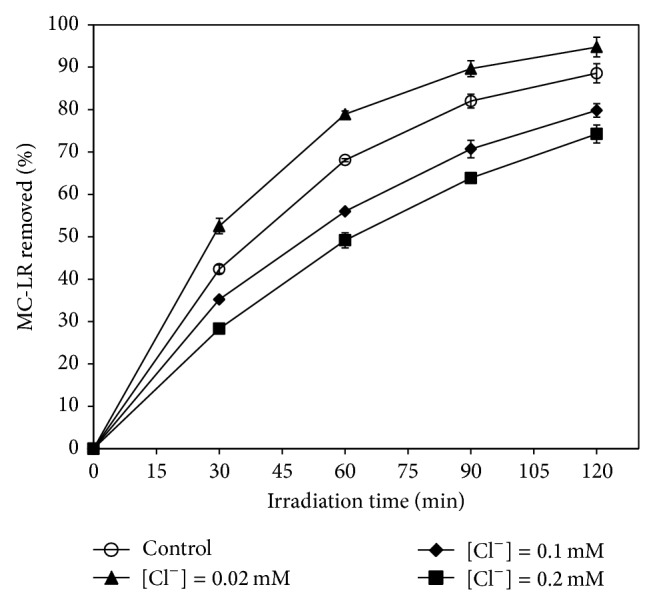
Efficiency of photocatalytic degradation of MC-LR as a function of Cl^−^ concentration. (Experimental conditions: MC-LR concentration of 1 mg L^−1^, Pt/WO_3_ concentration of 100 mg L^−1^, and simulated solar light intensity of 0.4 mW cm^−2^.)

Sodium chloride (NaCl) was introduced into the reaction solution at different concentrations to investigate the effect of Cl^−^ on the photocatalytic degradation of MC-LR. As shown in Figure 7, without Cl^−^ addition, about 88.6% MC-LR was removed after 120 min solar light irradiation. With Cl^−^ addition at the concentration of 0.02 mM, the percentage removal of MC-LR increased to 94.8%, whereas the percentage removal of MC-LR decreased to 79.8% and 74.2%, when the Cl^−^ concentrations were 0.1 mM and 0.2 mM, respectively. The results indicate that Cl^−^ at proper concentration could enhance the photocatalytic degradation of MC-LR, whereas excessive Cl^−^ could inhibit the degradation. This phenomenon can be ascribed to the formation of Cl radicals (∙Cl) in the photocatalytic reaction system. With Cl^−^ addition at an appropriate concentration, the photogenerated holes on the catalyst surface were scavenged by the Cl^−^ ions to form ∙Cl radicals [30, 31]. The ∙Cl radical is also a kind of high reactive species that can oxidize many organic substances. Guo et al. reported that Cl^−^ ions adsorbed on TiO_2_ surface promoted the photocatalytic oxidation of propylene [32]. However, excessive Cl^−^ ions can also scavenge ∙OH radicals to form Cl_2_ molecules very quickly, and the reactivity of Cl_2_ was lower than that of ∙OH [33]. Consequently, when adding Cl^−^ at an excessive concentration, the Cl^−^ ions began to scavenge ∙OH radicals preferentially that decreased the photocatalytic degradation of MC-LR.

### 3.6. Effect of Metal Cations (Cu^2+^ and Fe^3+^) on the Photocatalytic Degradation of MC-LR

The fast recombination of photogenerated electrons and holes on the catalyst surface is an important factor that limits the photocatalytic degradation of organic substances. Consequently, to enhance the photocatalytic activity of catalysts, improving the separation of photogenerated electron-hole pairs is very essential. Metal cations can be used as the scavengers of photogenerated electrons and seem to be an effect additive for suppressing the recombination of photogenerated electrons and holes. The enhanced photocatalytic activity of WO_3_ by addition of Cu^2+^ and Fe^3+^ in the reaction solution has been reported for the degradation of various organic substances such as phenol and sucrose [34, 35].

**Figure 8:**
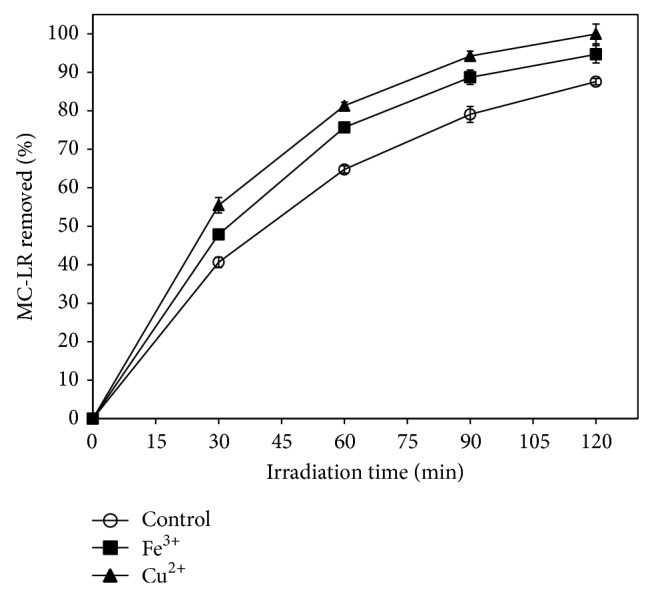
The effect of 0.2 mM of metal ions on the efficiency of photocatalytic degradation of MC-LR. (Experimental conditions: MC-LR concentration of 1 mg L^−1^, Pt/WO_3_ concentration of 100 mg L^−1^, and simulated solar light intensity of 0.4 mW cm^−2^.)

In order to investigate the effects of metal cations on photocatalytic degradation of MC-LR, Cu(NO_3_)_2_ and Fe(NO_3_)_3_ were introduced into the reaction solutions at a concentration of 0.2 mM. As shown in Figure 8, without metal cations addition, about 87.6% MC-LR was removed after 120 min solar light irradiation. In the presence of 0.2 mM Cu^2+^ and Fe^3+^, the percentage removal of MC-LR increased to 100% and 94.7%, respectively. The addition of Cu^2+^ and Fe^3+^ obviously enhanced the photocatalytic degradation of MC-LR under solar light irradiation.

The possible mechanism for the enhanced photocatalytic activity of Pt/WO_3_ by Cu^2+^ and Fe^3+^ addition can be described as follows. (1) The consumption of photogenerated electrons by the reduction of Cu^2+^ and Fe^3+^ ions suppressed the recombination of electrons and holes that increased the number of ∙OH radicals in the reaction system (7) and (8) [36]. In addition, (2) the Cu^2+^ and Fe^3+^ can react with H_2_O_2_ generated in a photo-Fenton reaction to produce additional ∙OH radicals in the reaction system (9) and (10). Consider

(7) $$\begin{array}{c} {\text{Cu}}^{2+}+{\text{e}}^{-}⟶{\text{Cu}}^{+} \end{array}$$

(8) $$\begin{array}{c} {\text{Fe}}^{3+}+{\text{e}}^{-}⟶{\text{Fe}}^{2+} \end{array}$$

(9) $$\begin{array}{c} {\text{Cu}}^{+}+{\text{H}}_{2}{\text{O}}_{2}+{\text{H}}^{+}⟶{\text{Cu}}^{2+}+∙\text{OH}+{\text{HO}}^{-} \end{array}$$

(10) $$\begin{array}{c} {\text{Fe}}^{2+}+{\text{H}}_{2}{\text{O}}_{2}+{\text{H}}^{+}⟶{\text{Fe}}^{3+}+∙\text{OH}+{\text{HO}}^{-} \end{array}$$

Then, the increased number of ∙OH radicals in the reaction solution promoted the photocatalytic degradation of MC-LR [37, 38]. According to Irie et al. [39] and Liu et al. [40], electrons in the surface grafted Fe^3+^ and Cu^2+^ ions efficiently cause multielectron reduction of adsorbed O_2_ molecules to achieve high quantum efficiency value. Therefore, the H_2_O_2_ produced during the multielectron reduction of O_2_ molecules also promoted the photodegradation of MC-LR in aqueous solution.

### 3.7. Photocatalytic Degradation Pathway of MC-LR

**Figure 9:**
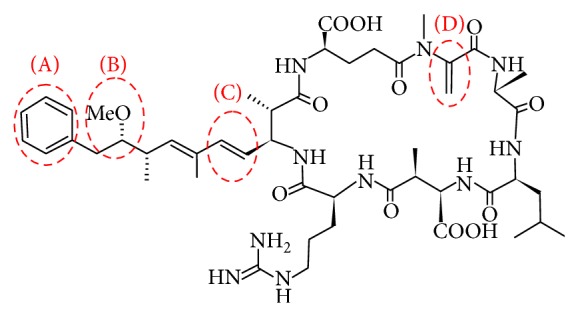
The molecular structure of microcystin-LR and the main attack sites ((A) benzene ring; (B) methoxy group; (C) conjugated double bond; (D) unsaturated double bond of MDHA) of hydroxyl radicals during photocatalytic reaction. ADDA: 3-amino-methoxy-10-phenyl-2,6,8-trimethyl-deca-4,6-dienoic acid.

The degradation pathway of MC-LR through photocatalytic reaction has been in detail reported by Su et al. [41]. As shown in Figure 9, MC-LR is a relatively large molecule with a cyclostructure, which consists of a usual 20-carbon amino acid (ADDA) that expresses biological toxicity and an amino acid N-methyldehydroalanine (MDHA). The MC-LR molecule is more readily attacked by ∙OH radicals at four sites of the toxin: three on the ADDA chain ((A) aromatic ring, (B) methoxy group, and (C) conjugated double bonds) and one on the cyclic structure ((D) MDHA amino acid) [28]. Among these, the conjugated double bond (site (C)) at the ADDA moiety of MC-LR molecule has been reported to be susceptible to photocatalytic attack [42, 43]. The destruction of MC-LR molecule by the attack of ∙OH radicals on these sensitive sites leads to production of many kinds of intermediate products, which can be degraded to final products by further reaction with ∙OH radicals.

In this present study, although the complete removal of MC-LR was obtained after 180 min solar irradiation when using Pt/WO_3_ as photocatalyst, less than 50% of the total MC-LR was mineralized. This can be attributed to the production of many kinds of intermediates which are stable against photocatalytic destruction and do not undergo complete oxidation. Since MC-LR was not completely mineralized, it is important to confirm that the intermediate products are nontoxic. Lawton et al. [14] assessed the toxicity of intermediates produced in photocatalytic degradation of MC-LR using brine shrimp bioassay method, and they could not detect any measureable toxicity.

## 4. Conclusions

A series of advanced WO_3_-based photocatalysts including CuO/WO_3_, Pd/WO_3_, and Pt/WO_3_ were developed for the photocatalytic removal of microcystin-LR (MC-LR) under simulated solar light irradiation. In this present study, when doped with CuO, Pd, and Pt, the generation rate of ∙OH radicals on WO_3_ surface was obviously enhanced. Pt/WO_3_ achieved the highest generation rate of ∙OH radicals and exhibited the best photocatalytic performance for the degradation of MC-LR under solar light irradiation. The photocatalytic degradation of MC-LR by Pt/WO_3_ under solar light well followed the pseudo-first-order kinetic equation. Cl^−^ addition at an appropriate concentration could enhance the photocatalytic degradation of MC-LR by Pt/WO_3_ under solar light irradiation. The addition of Cu^2+^ and Fe^3+^ obviously enhanced the photocatalytic degradation of MC-LR under solar light irradiation. The developed Pt/WO_3_ is a promising photocatalyst for enhancing the photocatalytic removal of recalcitrant organic compounds like MC-LR in water under solar light irradiation.
